# Dental Students’ Perception and Preference to Pediatric Dentistry Training

**DOI:** 10.1155/ijod/3246911

**Published:** 2026-02-26

**Authors:** Mais M. Almaeeni, Suhair W. Abbood Al-Osaighari, Eman A. Jaffer, Faaiz Y. Alhamdani

**Affiliations:** ^1^ Dentistry College, lbn Sina University of Medical and Pharmaceutical Sciences, Baghdad, Iraq, ibnsina.edu.iq; ^2^ POP Department, College of Dentistry, University of Anbar, Ramadi, Alanbar, Iraq, uoanbar.edu.iq

**Keywords:** behavior management, dental students, local anesthesia, pediatric dentistry

## Abstract

Pedodontics, or pediatric dentistry, is recognized as one of the most stressful fields within the health professions. Dentists need to create an environment that effectively manages children’s behaviors during dental treatments. This study aims to evaluate how undergraduate dental students perceive their training in pediatric dentistry and the challenges they face. To achieve this aim, a self‐designed questionnaire focusing on students’ attitudes toward pediatric dentistry was administered to final‐year students at seven universities in Iraq. The results indicated that students generally find pediatric dentistry to be quite challenging. According to their feedback, this difficulty stems primarily from behavior management issues, particularly related to children’s cooperation and their reluctance to accept local anesthesia. Despite these challenges, approximately one‐quarter of the respondents still expressed an interest in pursuing pediatric dentistry as their speciality in the future, with a notably higher number of female students showing this inclination compared to their male counterparts. Overall, the study concluded that pediatric dentistry is a demanding clinical practice, therefore, creating a proactive clinical environment that reduces stress and fosters positivity could lead to better treatment outcomes.

## 1. Introduction

Pediatric dentistry is a speciality focused on providing preventive and therapeutic oral healthcare for infants, children, and adolescents, including those with special healthcare needs [1]. One of the most important and critical aspects of pediatric dentistry is behavior guidance, which is essential for successfully performing dental treatments on children. Furthermore, children’s dental treatment is considered a one‐to‐two relationship among the dentist, patient, and parents, whereas for adults, it is a one‐to‐one relationship. These significant differences between child and adult treatment establish pediatric dentistry as having unique characteristics among other dental subjects. Thus, dental health professionals must be fully equipped and willing to create an environment that helps manage children’s behavior during these procedures, allowing them to enjoy their dental experiences, regardless of their initial attitudes [2–4].

This makes pediatric dentistry the most stressful among dental specialities, in addition to the fact that dental practice itself is often considered one of the most stressful professions, primarily due to the nature of clinical work, dealing with anxious patients, an unfriendly working environment, time constraints, and the need for continuous education to stay updated with advancements in the field [5, 6].

This stress can be amplified for undergraduate students, who face multiple assignments, competition with peers, and fear of failure. They also contend with strained relationships with faculty and fellow students, alongside the pressure of having to complete complex treatments while managing patient interactions [7, 8]. Furthermore, the levels of stress and anxiety among dental students may be even higher in pediatric dentistry due to the challenges associated with managing children’s behaviors. The treatment of children is influenced by what can be described as the pediatric dentistry triangle, which includes the child patient, the parents, and the dental team. Therefore, dental students should possess a solid understanding of behavior management techniques and pediatric treatment principles to form positive relationships with child patients, which is essential for effective dental care. Yes, research indicates that many dental students experience high levels of stress, particularly in pediatric clinics, where they often feel inadequately prepared to manage anxious children [7, 9, 10].

Since the final‐year dental students will soon join the medical workforce and healthcare system, they must acquire comprehensive knowledge and skills in pediatric dentistry. In Iraq, this is achieved by a combination of theoretical lecture curriculum and clinical training requirements, which are determined by the national scientific committee. The training starts in the fifth year. Although these national curricula exist, the clinical training varies between universities. The clinical requirements for pedodontics are included with variety of tooth therapies for both permanent and primary teeth, including restorative treatment, pulp therapy, extraction, and space maintainers, for children from age 4–12 years old.

This study was conducted to highlight challenges faced by final‐year dental students. These may be clinical competence, behavior management training, patient–parent interaction, variable supervision, and difficulties in meeting departmental requirements, which are considered an integral component of pediatric dentistry training. Since the students’ perceptions provide a significant insight about these challenges. This study aims to assess undergraduate dental students’ perceptions on pediatric dentistry training and the challenges they face, and identify educational gaps that could inform curriculum improvement. Furthermore, this study aims to assess pediatric dentistry preference as a speciality in the future.

## 2. Method

### 2.1. Study Design and Setting

The study consisted of a self‐designed questionnaire about students’ attitudes toward pediatric dentistry. This cross‐sectional study included final‐year dental students from different public and private universities in Iraq. Incomplete responses or responses from other students not in their final‐year were excluded. Cluster random sampling was employed, with universities being randomly selected as the primary sampling units. This study includes all the final‐year students from seven universities in different Iraqi governorates to capture a broad and representative range of student experiences. This enhances the generalizability of the finding and warrants for a comprehensive evaluation of pediatric dentistry at the national level. The data presented in the tables were collected from December 2022 to March 2023.

### 2.2. Ethical Approval

Ethical Approval Number ISU.12.1.24 was obtained from the Institutional Review Board (IRB)—Ethical Committee at Ibn Sina University of Medical and Pharmaceutical Sciences. The protocol designed for this study was approved by the Scientific Committee/College of Dentistry/Ibn Sina University of Medical and Pharmaceutical Sciences.

### 2.3. Sample Size Calculation

The sample size was calculated by a proportion‐based formula using an online sample size calculator (Calculator.net). The calculated sample size was 363, with a confidence level of 95% and a margin of error of 5%, based on a total population of 6418, according to data obtained from the Ministry of Education, Iraq. The sample size was subsequently increased to 513 to reduce the margin of error to 4.07%.

### 2.4. Questionnaire Development

This questionnaire was based on an online focus group discussion. Eight final‐year undergraduate students from the College of Dentistry, Al‐Mustansiriyah University, agreed to participate in a Zoom meeting, which lasted for 1 h and 29 min, to explore their views on different aspects of pediatric dentistry subject areas. The students were informed that their opinions would be anonymized, and they expressed their consent. The Zoom meeting was recorded and transcribed verbatim using thematic analysis of the data to identify the key codes and themes. The identified codes and themes were considered in the formulation of the study questionnaire (Table 1). Additionally, the questionnaire’s design was significantly influenced by the research team’s 25 years of accumulative educational experience.

**Table 1:** Themes and codes that used for questionnaire development.

Codes	Themes
What does pediatric dentistry need?	What does pedodontics mean to you?
How pediatric dentistry differs from other dental specialities?	
The role of parents	
Gender suitability for this speciality	

Difficulty of the first encounter	Difficulty in pediatric dentistry
Difficulty in dealing with very small children	

The presence of a previous relationship	First encounter with the child
How did you deal with the child?	
Have you followed special tactics?	

From lectures, and from which syllabus	Information availability
The need for psychology lectures	
The need for another information	

The tutor	Challenges in pediatric dentistry
Shortage in patients	
Difficulty of clinical sessions	
Sense of accomplishment	

A pilot study was conducted where the questionnaire was reviewed by 10 students for face validity. For content validity, six pediatric dentist specialists assessed each questionnaire item’s relevance, then the content validity test (CVT) was calculated.

### 2.5. Statistical Analysis

The data were analyzed using the Statistical Package for the Social Sciences (SPSS Version 26, IBM, USA). Descriptive statistics, including frequencies and percentages, were calculated for each questionnaire item. A chi‐square test was performed to assess the association between gender and preference of pediatric dentistry as their future speciality.

## 3. Results

The individual‐content validity index (I‐CVI) and scale‐CVI (S‐CVI) were calculated. I‐CVI was 0.83 and S‐CVI was 0.85, indicating strong content validity. Furthermore, the modified kappa coefficient was calculated as 0.816, demonstrating an excellent level of agreement among the experts. For better reliability of the instrument, the questionnaire had been tested by Cronbach’s *α*, which was 0.729. In addition to that, its development was based on qualitative data. Fifth‐stage dentistry students participated in this study. The sample size was 513 (138 males and 375 females), as revealed in Table 2.

**Table 2:** Sample distribution according to gender.

Gender	No.	%
Male	138	26.9
Female	375	73.1
Total	513	100

Table 3 illustrates student perceptions of the difficulty of pediatric dentistry relative to other specialities and the factors that make pediatric patient management difficult. As shown in Table 3, the majority of students found pediatric dentistry difficult to extremely difficult. Most of the students believed that the factors that make pediatric patient management difficult were uncooperative children and refusal of local anesthetic injection.

**Table 3:** Questionnaire answer frequencies.

Questions	No.	%
The difficulty of pediatric dentistry among other specialities		
Not difficult	9	1.76
Slightly difficult	49	9.55
Difficult	221	43.08
Very difficult	209	40.74
Extremely difficult	25	4.87
Factors that make pediatric patient management difficult		
Uncooperative children	308	60.04
Refusal of local anesthetic injection	123	23.98
Lack of tutor support when needed	17	3.31
The sound of the dental turbine/handpiece	14	2.73
Young children (5 years or less)	51	9.94
How do students rate the usefulness of theoretical lectures in treating a child?		
Not useful at all	9	1.8
Of little use	61	11.9
Useful to a certain extent	249	48.5
Very useful	149	29
Extremely useful	45	8.8
Parents’ level of education helps in child management		
Do not agree	2	0.39
Agree to some extent	19	3.7
Agree	106	20.66
Strongly agree	123	23.98
Extremely agree	263	51.27
The benefit of parents’ presence in the clinic		
Not useful at all	9	1.8
Of little use	0	0
Useful sometimes	247	48.1
Very useful	182	35.5
Extremely useful	75	14.6
To what extent do students advise the presence of the parents of young children (5 years or less)?		
I do not recommend the parents’ presence	6	1.2
If their role is positive	87	17
During a certain part of the procedure	112	21.8
Most of the time	115	22.4
Always	193	37.6
To what extent do you think that psychology subjects are important for child management?		
Not important	0	0
Of little importance	0	0
Important	143	27.9
Very important	180	35.1
Essential	190	37
Do students agree with the fact that female students are more capable of child dental management than males?		
I do not agree	23	4.5
Agree to a little extent	98	19.1
Agree to a certain extent	180	35.1
Agree to a high extent	142	27.7
Extremely agree	70	13.6

The vast majority of students, comprising those who “extremely agreed,” “strongly agreed,” and “agreed,” believed that parents’ level of education positively influenced child management during dental procedures. Additionally, 50% of students rated parents’ presence in the dental clinic as very useful or extremely useful. More than half of dental students recommended that parents should always or most of the time be in the clinic for children aged 5 years old or less.

Regarding the perceived importance of psychological principles in child management (how the students rate the importance of the psychology of the subject in child management and the usefulness of the theoretical literature in child management), all students rated it as “important,” “very important,” or “essential.” Furthermore, concerning the usefulness of theoretical lectures, nearly half of the students perceived them as “useful to a certain extent,” while a small percentage considered them “not beneficial at all.” The final question in Table 3 explored students’ opinions on the perceived capability of female students to manage children’s dental patients compared to males. In general, responses indicated a tendency toward agreement with female capability in this regard.

Results revealed a highly significant association between gender and selection of pediatric dentistry as their future profession. About 31% of female students expressed a preference for pediatric dentistry as their future speciality, compared to 14% of male students, as shown in Table 4.

**Table 4:** Frequency of student who prefer pediatric dentistry as their speciality in the future and its relation to gender.

Students’ preference to pediatric dentistry as their speciality in the future			
	Yes	No	
Gender			Chi‐square test *p* ~ 0.001 df = 1 Highly significant (*p* < 0.01)
Male	19	119	
Female	117	258	

## 4. Discussion

Students’ views on the learning process are considered a cornerstone in the undergraduate education process [11, 12]. The current study revealed that over 70% of the respondents were females, which aligns with expectations (Table 2). This indicates that females constitute a larger proportion of dental professionals in Iraq. Similar findings are consistent with findings from other dental schools in the Middle East [13, 14].

Table 3 demonstrates students’ responses, begin with “The difficulty of pediatric dentistry among other specialities.” It is evident from the student’s perspective that pediatric dentistry is a challenging discipline. This may be related to students’ lack of experience with child behavior management. Since there is no prior clinical training in the fourth year, unlike other dental subjects [4, 7, 15].

Challenges in dental training in general have been widely discussed in the literature. Furthermore, child behavior management presents more complexities. These include the application of theoretical knowledge in clinical settings in the clinical environment, child behavior management, training clinic atmosphere, local anesthesia application, the presence of parents, and children aged 5 years and younger. It has more than one aspect [1, 7, 16]. Participants primarily cited children’s uncooperativeness as a challenge, with refusal of local anesthesia injection being the second most frequent concern. Poor child management control by the dental student suggests a role in this aspect. Students seem to demonstrate difficulty in effectively applying the instructions provided in theoretical lectures. Interestingly, student respondents in this study stated that their lectures were useful to a certain extent. This may reflect inadequate theoretical acquisition by the student. These findings highlight the necessity of integrating child behavior management more explicitly into the evaluation criteria for clinical training.

Child management seems to be influenced by a multitude of factors. From the student’s perspective, parents’ education and presence during treatment are influential factors. These have been found to be influential in child management in pediatric dentistry, particularly in preschool children [17–19]. A clinical atmosphere might be another reason. Specifically, student anxiety stemming from clinical requirements may influence children’s behavior [19, 20].

Participants generally agreed that studying child psychology would positively influence child behavior management in the dental clinic. Theoretical material addresses some of the psychological aspects of child management [3]. Therefore, incorporating child psychology material into the dental curriculum warrants further research.

Students’ career choices appear to be influenced by perceived career challenges, which may explain the limited preference for pediatric dentistry among participants, as shown in Table 4. Nevertheless, female students are more willing to consider pediatric dentistry as their future career, a perception affirmed by both genders. Maternal instinct could be a contributing factor. Also, social expectation, cultural base, and patient–family dynamics in Iraq may affect how males and females select their speciality. This emphasizes the need for studies to investigate gender‐related trends in dental education [13, 21].

However, the study has its limitations and suggests that future studies should triangulate findings by incorporating faculty evaluations or objective performance assessments to provide a more comprehensive understanding of students’ clinical preparation. Also, suggest a study to assess whether attitudes change with increased clinical experience or postgraduation, which may afford deeper knowledge about how clinical exposure influences attitude toward pediatric dentistry.

## 5. Conclusion

Pediatric dentistry is a challenging clinical practice. Fostering a proactive clinical environment that emphasizes stress reduction and a positive clinical atmosphere could lead to improved treatment outcomes. These findings provide practical and evidence‐based guidance of curriculum improvement to meet student’s educational needs. This can be achieved by the integration of child psychology education in theoretical lectures, behavior management training, improved supervision strategies, and structured communication skills modules.

## Author Contributions

Study design and concept development by Faaiz Y. Alhamdani. Sampling strategies and data collection and analysis performed by Mais M. Almaeeni. Result verification and draft writing performed by Mais M. Almaeeni and Eman A. Jaffer Al‐Timimi. Revision manuscript draft and qualitative interview analysis performed by Faaiz Y. Alhamdani and Suhair W. Abbood Al‐Osaighari.

## Funding

This research did not receive any external funding, grants, or other support during the preparation of this manuscript.

## Disclosure

All authors read and approved the final manuscript.

## Conflicts of Interest

The authors declare no conflicts of interest.

## Data Availability

The data that support the findings of this study are available from the corresponding author upon reasonable request.
